# Establishment of RpHluorin2-expressing cell and its application in monitoring JTC-801-induced alkaliptosis via multi-dimensional fluorescence detection approaches

**DOI:** 10.3389/fcell.2025.1727740

**Published:** 2025-12-12

**Authors:** Jie Guo, Feng-Yi Mai, Xin-Yu Li, Jing-Rong Liang, Wen-Tao Yang, Chen-Guang Li

**Affiliations:** 1 Department of Rheumatology and Immunology, Shenzhen Second People’s Hospital, Shenzhen, China; 2 Shenzhen Nanshan People’s Hospital, Affiliated Nanshan Hospital of Shenzhen University, Shenzhen, China; 3 Southern University of Science and Technology, Shenzhen, China; 4 Department of Breast and Thyroid Surgery, Union Hospital, Tongji Medical College, Huazhong University of Science and Technology, Wuhan, China

**Keywords:** alkaliptosis, cytosolic pH, JTC-801, multi-dimensional fluorescence detection, RpHluorin2

## Abstract

Dysregulation of intracellular pH (pHi) is a hallmark biological feature of cancer cells, which hijack pHi homeostasis to sustain malignant phenotypes including uncontrolled proliferation, invasion, and metabolic reprogramming. Meanwhile, alkaliptosis, a newly defined pH-dependent form of regulated cell death specifically triggered by the small-molecule compound JTC-801, has emerged as a promising therapeutic target for cancer intervention. However, reliable tools for monitoring dynamic pHi changes during alkaliptosis remain insufficient. RpHluorin2 is an optimized ratiometric pH-sensitive green fluorescent protein variant with enhanced fluorescence intensity and stability. Herein, we established a stable RpHluorin2-expressing cell line and further developed a multi-dimensional fluorescence detection workflow, which encompasses a microplate reader, fluorescence microscopy, flow cytometry, a small animal imaging system (IVIS Spectrum), and a protein dot blot assay coupled with IVIS. Our results demonstrated a strong linear correlation between RpHluorin2 fluorescence intensity and cell number, with a coefficient of determination (*R* ^2^) of 0.9998. Furthermore, across all employed detection methods, JTC-801 treatment induced dose-dependent and time-dependent reductions in RpHluorin2 fluorescence, an observation that is consistent with the elevation of pHi during alkaliptosis. This platform enables high-throughput screening, single-cell analysis, and biochemical validation, providing a robust tool for mechanistic research on alkaliptosis and the development of pH-targeted anticancer therapies.

## 1 Introduction

Intracellular pH (pHi) serves as a pivotal regulatory mediator for a broad spectrum of biological functions, given that every cellular process depends on a precisely delineated pH range to sustain its state of optimal operational efficiency. Even subtle fluctuations in pHi can disrupt the regulatory dynamics of diverse molecular events, including the catalytic efficacy of enzymes, the gating kinetics of ion channels, and the substrate translocation efficiency of membrane transporters, all of which constitute essential molecular machineries that underpin the execution and maintenance of normal cellular functions (Romero-Moreno et al., 2024). The dysregulation of cellular metabolism represents a hallmark of cancer, with tumor microenvironment (TME) acidification emerging as a critical consequence and driver of malignant progression (Xu et al., 2022; Xu et al., 2025). Under aerobic conditions, normal cells primarily utilize oxidative phosphorylation as the core pathway for energy generation. In marked contrast, cancer cells exhibit a distinct metabolic reprogramming: they prioritize glycolysis for energy production, and this preferential shift remains evident even in the presence of ample oxygen (Chen et al., 2023a). This metabolic reprogramming leads to excessive production of lactic acid, which, when coupled with impaired acid extrusion mechanisms in the TME, results in a progressive reduction in extracellular pH (pHe) to values as low as 6.0–6.5, while pHi remains relatively neutral or slightly alkaline. Beyond metabolic reprogramming, the TME also shapes tumor progression through complex interactions among immune cells, cytokines and stromal components, as highlighted in recent studies (Sharma et al., 2015). This observation has therefore propelled the systematic exploration of pH modulation as a prospective therapeutic strategy in the context of cancer management (Webb et al., 2011; Ward et al., 2020).

Recent investigations have established that intracellular acidification within cancer cells is capable of inducing diverse forms of cell death, such as apoptosis and necroptosis (Webb et al., 2011). In comparison, alkaliptosis is a distinct form of regulated cell death driven by intracellular alkalization. First characterized and named by Daolin Tang in 2018, this pH-dependent non-apoptotic cell death process is triggered by JTC-801 (a small-molecule opioid analgesic compound) and has been identified as a novel strategy for malignant tumor therapy (Yamada et al., 2002; Song et al., 2018). Currently, JTC-801 serves as a prototypical alkaliptosis inducer. It departs from its well-characterized role as a selective pain peptide receptor antagonist; instead, it mediates alkaliptosis by activating nuclear factor kappa B (NF-κB). Notably, JTC-801-induced NF-κB activation downregulates the expression of carbonic anhydrase 9 (CA9), a molecule responsible for maintaining pH homeostasis, and this regulatory process ultimately triggers alkaliptosis accompanied by concurrent plasma membrane rupture (Yamada et al., 2002; Song et al., 2018; Liu et al., 2020; Chen et al., 2023b). As a cancer therapeutic strategy, alkaliptosis exhibits notable potential, given that cancer cells display marked pH dysregulation, and their proliferation, metastatic capacity, and metabolic adaptation are governed by their inherent pH sensitivity. Drugs developed to target alkaliptosis may thus emerge as a new approach to cancer therapy, especially for malignancies resistant to standard treatment modalities (Koltai, 2016; Liu et al., 2020). However, additional in-depth research is required to fully elucidate the molecular mechanism underlying alkaliptosis and its therapeutic potential in cancer treatment. Therefore, there is an urgent need to develop more drugs targeting alkaliptosis. A key characteristic of alkaliptosis is the elevation of intracellular pH (pHᵢ) (Song et al., 2018; Chen et al., 2023b; Chen et al., 2024). Consequently, the availability of reliable tools or methodologies for detecting cytosolic pH changes would significantly facilitate the research and development of alkaliptosis-targeted drugs, as well as their clinical translation.

The exploration of cellular processes governed by pH is hindered by constraints in experimental methodologies, with particular difficulties arising in the quantification of pH within living cells (Miesenbock et al., 1998). Even subtle perturbations in pHi can disrupt these molecular events, underscoring the necessity for robust tools to monitor pHi dynamically in living systems. However, quantifying pHi poses significant technical challenges, particularly in scenarios involving microscale cellular compartments (e.g., organelles) or morphologically small cells, where traditional pH-sensing approaches often fall short. To address these limitations, fluorescent pH indicators have emerged as indispensable tools, with ratiometric probes standing out for their ability to provide quantitative, background-corrected pH measurements. Ratiometric pHluorin2 (RpHluorin2), an enhanced ratiometric pH-sensitive green fluorescent protein variant developed using GFP2 as the template (Miesenbock et al., 1998; Bizzarri et al., 2006; Mahon, 2011). As an optimized variant, it typically exhibits enhanced fluorescence intensity, improved pH stability, and superior folding efficiency compared to its predecessors (native pHluorin) and related GFP variants, making it highly suitable for in live-cell imaging and real-time biological process monitoring (Mahon, 2011; Linders et al., 2022). RpHluorin2 emits green fluorescence upon excitation (with typical excitation and emission peaks depending on specific engineering, often in the range of 395–475 nm for excitation and 509 nm for emission, respectively) (Mahon, 2011).

In the present study, we established RpHluorin2-expressing cell models to monitor fluorescence alterations during alkaliptosis. Concurrently, we developed a suite of detection methodologies for RpHluorin2-derived fluorescence changes, encompassing microplate reader and fluorescence microscopy analyses, flow cytometry analyses, as well as small animal imaging system-based detection. Collectively, these RpHluorin2-expressing cells and multidimensional fluorescence detection approaches enable quantitative monitoring of JTC-801-induced alkaliptosis. Notably, the development of RpHluorin2-expressing cell models and the corresponding fluorescence detection strategies provides a robust technical platform for investigating the molecular mechanisms underlying alkaliptosis and facilitating the research and development of alkaliptosis-related novel therapeutic drugs.

## 2 Materials and methods

### 2.1 Reagents and antibodies

RpHluorin2 plasmid (#YM77317) was bought from Yanming Biotechnology (Shenzhen, China). JTC-801 (#HY-13274) was bought from MCE (Princeton, USA). Hoechst33342 (#B2261) were bought from Sigma. Fetal bovine serum, RPMI-1640 medium, BCA protein assay kit, and Lipofectamine 2000 were bought from Thermo Fisher (Carlsbad, United States). Polyvinylidene fluoride membrane (#FFP70), RIPA Lysis Buffer (#P0013B), PMSF (#ST506), puromycin (#ST551) and BCECF AM (#S1006) were obtained from Beyotime. pHrodo Green AM intracellular pH indicator (#P35373) was bought from Thermo Fisher Scientific.

### 2.2 Cell line culture

The human cell line 5637 (catalog number: CL-0002) was sourced from Procell (Wuhan, China). Cells were grown in complete RPMI-1640 medium (supplemented with 10% FBS) and incubated in a humidified environment with 5% CO_2_ at 37 °C. Subculture operations were carried out at intervals of 2–3 days.

### 2.3 Quantitative detection of intracellular pH

Intracellular pH was determined using a fluorogenic cytoplasmic pH indicator probe (Thermo Fisher Scientific). Fluorescence intensity of the probe is then an indicator of intracellular pH. This reagent can quantify cellular cytosolic pH in the range of four to nine with a pKa of∼6.5. Subsequent use of the Intracellular pH Calibration Buffer Kit (P35379) allows this intracellular pH to be quantified. Briefly, cells were washed with Live Cell Imaging Solution (LCIS), then 10 μL of pHrodo™ Green AM was mixed with 100 μL of PowerLoad™ concentrate and was add to 10 mL of LCIS. Cells were incubated with the pHrodo™ AM/PowerLoad™/LCIS at 37 °C for 30 min and then were quantified using a microplate reader (Bio-Rad, USA).

### 2.4 Exogenous expression of RpHluorin2 in cancer cells

RpHluorin2, a pH-sensitive green fluorescent protein variant widely used to monitor cytosolic pH dynamics, was exogenously expressed in human bladder cancer 5637 cells via lentiviral transduction to establish a stable cell line. The RpHluorin2 plasmid was synthesized by Yanming Biotechnology company (Shenzhen, China). The recombinant plasmid (pLV3-CMV-RpHluorin2-Puro) was transformed into DH5α competent *Escherichia coli*, and positive clones were screened by ampicillin resistance (100 μg/mL) and verified via Sanger sequencing (Sangon Biotech, shanghai, China) to confirm the correct insertion and sequence integrity of RpHluorin2. For lentivirus production, HEK293T cells were seeded in 10-cm dishes at 5 × 10^6^ cells/dish and cultured in DMEM supplemented with 10% FBS under 37 °C and 5% CO_2_. At 70%–80% confluence, cells were co-transfected with 10 μg recombinant plasmid, 7.5 μg packaging plasmid psPAX2, and 3 μg envelope plasmid pMD2. G using Lipofectamine 2000. Transfection medium was replaced with fresh complete DMEM after 6 h. The lentiviral supernatant was collected at 48 h and 72 h post-transfection, filtered through a 0.45 μm polyethersulfone filter, and concentrated via centrifugation at 4000 rpm for 10 min at 4 °C. 5637 cells were seeded in 6-cm dishes and cultured to 50%–60% confluence. The medium was removed by aspiration, and replaced with 3 mL of fresh complete DMEM supplemented with 1 mL of the RpHluorin2 lentivirus and 6 μg/mL polybrene to enhance viral transduction efficiency. At 48 h post-transduction, cells were trypsinized and reseeded into new 6 cm dishes, and stable cell clones were selected by puromycin. Following 7–10 days of sustained selection, further experiments were performed to expand the scope of the investigation.

### 2.5 Microplate reader-based detection of intracellular fluorescence

RpHluorin2-expressing cells were seeded into 96-well black-walled clear-bottom microplates (Corning, USA) at a density of 1 × 10^4^ cells per well in 100 µL of RPMI1640 complete culture medium. For experimental groups, RpHluorin2-expressing cells were treated with JTC-801 at concentrations of 10, 20, and 40 μM for 6 h, while a separate set of cultures was exposed to 40 μM JTC-801 for 1, 3, and 6 h, respectively. Intracellular fluorescence was quantified using a microplate reader (Bio-Rad, USA). The microplate reader was used to quantify and record fluorescence intensity, where excitation was set to 479 nm and emission to 520 nm. Background fluorescence, measured from wells containing non-fluorescent control cells, was subtracted from sample values. Each measurement was conducted in three independent biological replicates to ensure experimental reliability.

### 2.6 Fluorescence microscopy and imaging

RpHluorin2-expressing cells were seeded in a 24-well plate, with a seeding density of 5 × 10^4^cells per well. Subsequently, cells were treated with JTC-801 at the indicated concentrations for the specified time periods. The cells were stained with Hoechst33342 solution (5 μg/mL) for 10 min at room temperature and observed immediately by live imaging using Zeiss Axio Observer D1 microscope (Carl Zeiss MicroImaging GmbH, Göttingen, Germany). Median fluorescence intensity values were analyzed via ImageJ software (National Institutes of Health, Bethesda, MD, USA) following standard protocols.

### 2.7 Flow cytometry analysis

RpHluorin2-expressing cells were treated with JTC-801 at the indicated concentrations for the specified time periods. Adherent cells were gently detached using trypsin at 37 °C for 2–3 min. Detached cells were collected into 15 mL sterile centrifuge tubes, and the remaining adherent cells were thoroughly rinsed with pre-warmed PBS to ensure complete recovery. The cell suspension was then supplemented with an equal volume of complete RPMI1640Medium containing 10% fetal bovine serum to neutralize trypsin activity. The harvested cell suspension was centrifuged at 1,200×g for 5 min at 4 °C to pellet the cells, and the pellet was then resuspended in 2 mL of ice-cold PBS. Data were acquired on a flow cytometer equipped with CELLQuest software. For each sample, at least 20,000 events were acquired to ensure statistical reliability. Unexpressed RpHluorin2 cells were used to establish autofluorescence baseline. Each experimental group was analyzed in triplicate. The median fluorescence intensity was analyzed by the FlowJo 10 software.

### 2.8 Fluorescence imaging via a small animal imaging system

RpHluorin2-expressing cells were seeded into 96-well black-walled clear-bottom microplates (Corning, USA) at a density of 1 × 10^4^ cells/well. Subsequently, cells were treated with JTC-801 at the indicated concentrations for the specified time periods. Fluorescence imaging was performed using a small animal *in vivo* fluorescence imaging system (IVIS Spectrum, PerkinElmer, Waltham, MA, USA) to visualize and quantify the fluorescence of RpHluorin2. After parameter confirmation, fluorescence images were acquired in radiant efficiency units to enable quantitative comparison across samples. Image analysis was conducted using ZEN Image Software. All imaging was performed in a dark room to prevent ambient light from interfering with fluorescence detection.

### 2.9 Protein dot blot assay

After treatment, RpHluorin2-expressing cells were washed twice with ice-cold PBS, then lysed in lysis buffer supplemented with 100 μM PMSF on ice for 30 min. Cell lysates were centrifuged at 12,000×g for 10 min at 4 °C to eliminate insoluble debris fractions The supernatant (soluble protein fraction) was collected into pre-chilled microcentrifuge tubes. Protein concentration was determined using a BCA protein assay kit following the manufacturer’s protocol to ensure uniform loading. Sample was spotted onto a PVDF membrane (0.22 μm pore size) using a custom-developed spotting apparatus for protein dot blot. The membrane was air-dried at room temperature for 20 min to fix proteins. For semi-quantitative analysis, the PVDF membranes containing the samples were transferred to a small animal imaging system (IVIS Spectrum) for fluorescence detection. IVIS Spectrum *in vivo* imaging system equipped with a high-sensitivity cooled CCD camera, which enabled quantitative detection of RpHluorin2 fluorescence signals. Each measurement was conducted in three independent biological replicates to ensure experimental reproducibility.

### 2.10 Statistical analysis

Each experiment was conducted with three biological replicates. Data were expressed as mean ± SD, and statistical analyses to determine significance were performed using GraphPad Prism 9.0 (San Diego, CA, United States). One-way ANOVA was employed to analyze statistical differences among multiple groups, with *p* < 0.05 considered indicative of statistical significance.

## 3 Results

### 3.1 Establishment of RpHluorin2-expressing cells and fluorescence detection via diverse methodologies

**FIGURE 1:**
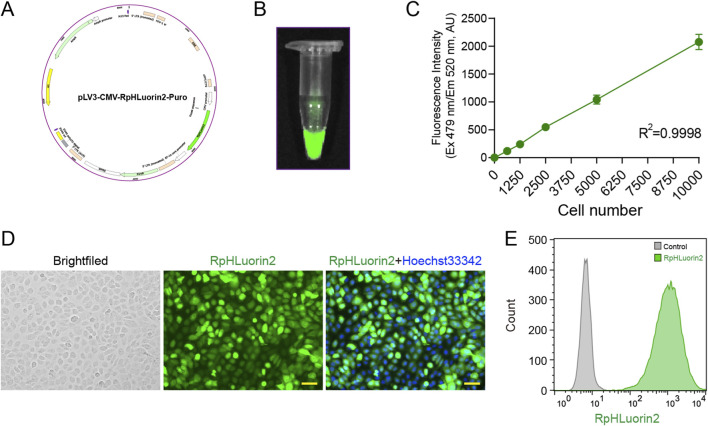
Establishment of RpHluorin2-expressing cells and fluorescence detection using multiple detection methodologies. **(A)** Schematic map of the RpHluorin2 expression plasmid. **(B)** Fluorescence images of RpHluorin2-expressing cells captured by small animal imaging system. **(C)** Linear correlation between fluorescence intensity and cell number. RpHluorin2-expressing cells were seeded at varying densities into 96-well black-walled clear-bottom microplates and incubated overnight. The microplate reader was used to quantify and record fluorescence intensity, where excitation was set to 479 nm and emission to 520 nm. **(D)** Representative fluorescence images showing RpHluorin2 (green) subcellular expression were captured by fluorescence microscopy. Nuclei (blue) were revealed by Hoechst33342. Scale bars, 50 μm. **(E)** The fluorescence intensity of RpHluorin2-expressing cells was analyzed using flow cytometry. The gray histogram represents the control group (non-expressing cells), and the green histogram represents RpHlurin2-positive cell.

RpHluorin2, a pH-sensitive green fluorescent protein variant widely employed for monitoring cytosolic pH dynamics (Mahon, 2011). We aim to establish RpHluorin2-expressing cells for monitoring fluorescence changes during alkaliptosis. The construction of RpHluorin2-expressing cells and the subsequent fluorescence detection were performed with multiple detection methodologies. Specifically, the RpHluorin2-encoding gene was cloned into an appropriate expression vector and then transfected into cells using an optimized transfection method to achieve robust and consistent expression of RpHluorin2. The plasmid map of RpHluorin2 provided the genetic blueprint (Figure 1A; Supplementary Figure S1). Fluorescence imaging via a small animal imaging system revealed robust green fluorescence emission from the RpHluorin2-expressing cells (Figure 1B). To examine the linear relationship between fluorescence intensity and cell number, RpHluorin2-expressing cells were seeded at varying densities into microplates and incubated overnight. Subsequently, a microplate reader was used to measure and record fluorescence intensity, with excitation wavelength set at 479 nm and emission wavelength at 520 nm. The resulting scatter plot showed an excellent linear relationship between fluorescence intensity and cell density, as indicated by a high correlation coefficient (R^2^ = 0.9998, Figure 1C), highlighting the reliability of using fluorescence intensity to reflect cell number. Fluorescence microscopy imaging further visualized the subcellular expression of RpHluorin2 (green). These results demonstrated that under resting conditions, the intracellular green fluorescence of the cells was remarkably prominent (Figure 1D). Flow cytometry analysis was conducted to assess fluorescence intensity at the single-cell level. The gray histogram represented the control group without RpHluorin2 expression, while the green histogram corresponded to RpHluorin2-expressing cells, clearly demonstrating the distinct fluorescence profile of the engineered cells (Figure 1E). Collectively, these results confirm the successful establishment and reliable fluorescence properties of RpHluorin2-expressing cells, laying a foundation for their application in monitoring alkaliptosis-related processes.

### 3.2 Microplate reader and fluorescence microscopy analyses reveal dose-and time-dependent reduced RpHluorin2 fluorescence in JTC-801-induced alkaliptotic cells

**FIGURE 2:**
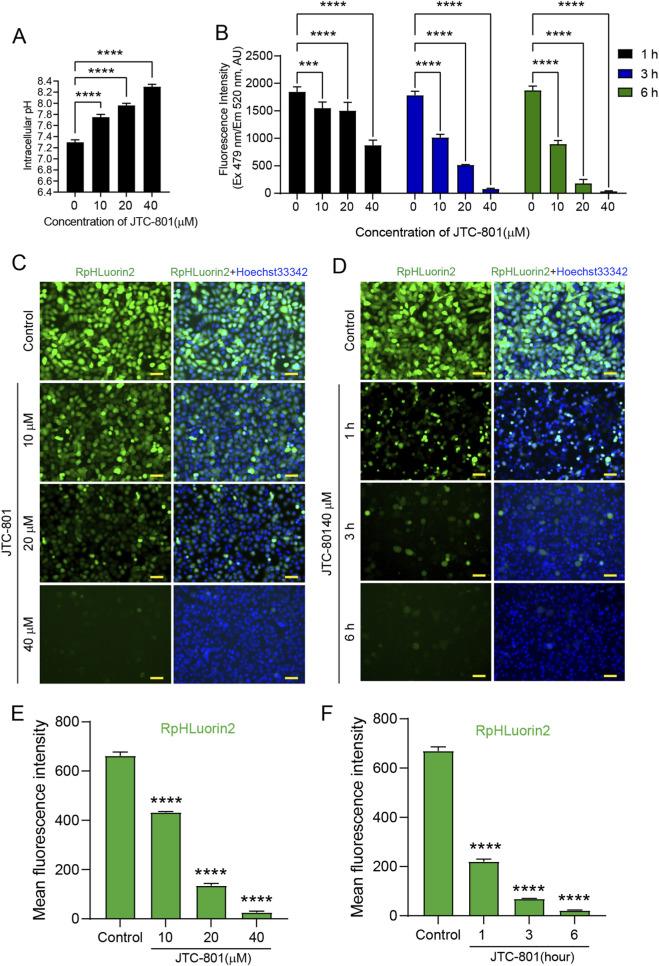
Microplate reader and fluorescence microscopy analyses showed that JTC-801 induces dose-and time-dependent reduced RpHluorin2 fluorescence in alkaliptotic cells. **(A)** JTC-801 was administered to cells at concentrations of 10, 20, and 40 μM for 6 h, and intracellular pH was subsequently assayed. **(B)** RpHluorin2-expressing cells were treated with JTC-801 at concentrations of 10, 20, and 40 μM for 6 h, while a separate set of cultures was exposed to 40 μM JTC-801 for 1, 3, and 6 h, respectively. The microplate reader was used to quantify and record fluorescence intensity, where excitation was set to 479 nm and emission to 520 nm ***p < 0.001; ****p < 0.0001. **(C,D)** Representative fluorescence images showing RpHluorin2 (green) subcellular expression were captured by fluorescence microscopy. Nuclei (blue) were stained with Hoechst33342. Scale bars, 50 μm. **(E,F)** Mean fluorescence intensity values of RpHluorin2 were measured and calculated via ImageJ software. ****p < 0.0001.

The small-molecule compound JTC-801 is a well-characterized inducer of alkaliptosis, a recently defined form of regulated cell death (Song et al., 2018). We first treated cells with JTC-801 and measured changes in intracellular pH. The results showed that JTC-801, as an inducer of alkaliptosis, significantly increased intracellular pH in a dose-dependent manner (Figure 2A). This observation is consistent with previous research reports (Song et al., 2018; Chen et al., 2023b; Chen et al., 2024). To investigate the effects of JTC-801 on RpHluorin2 fluorescence levels in alkaliptotic cells, we utilized a stably expressing RpHluorin2 cell model and subjected these cells to different treatments. As shown in Figure 2B, cells were treated with JTC-801 at concentrations of 10, 20, and 40 μM for 6 h, or exposed to 40 μM JTC-801 for 1, 3, and 6 h respectively. Fluorescence intensity quantified via a microplate reader revealed a dose-dependent reduction in RpHluorin2 signal under the concentration-gradient treatment, as well as a time-dependent decrease in the time-course experiment. Meanwhile, to further verify that the decrease in RpHluorin2 fluorescence is associated with an increase in intracellular pH, we also quantified pH using the ratiometric pH-sensitive fluorescent dye BCECF-AM, a well-established tool for pH monitoring. Consistently, we found that JTC-801 treatment dose-dependently reduced BCECF-AM fluorescence (Supplementary Figure S2). Complementary to these quantitative measurements, fluorescence microscopy imaging further confirmed a gradual attenuation of RpHluorin2 fluorescence with increasing JTC-801 concentrations and prolonged exposure times In the dose-dependent experiment, a significant reduction in the mean fluorescence intensity of RpHluorin2 was observed with increasing concentrations of JTC-801, as compared to the control group (Figures 2C,E). Similarly, in the time-dependent assay, when cells were exposed to a fixed concentration of 40 μM JTC-801, the mean fluorescence intensity of RpHluorin2 exhibited a prominent time-dependent decrease as the exposure duration was extended (Figures 2D,F). These results collectively indicate that JTC-801 induces both dose- and time-dependent reductions in RpHluorin2 fluorescence levels in alkaliptotic cells, which can be effectively visualized and quantified using microplate reader-based assays and fluorescence microscopy.

### 3.3 Flow cytometry analyses reveal dose-and time-dependent reduced RpHluorin2 fluorescence in JTC-801-induced alkaliptotic cells

**FIGURE 3:**
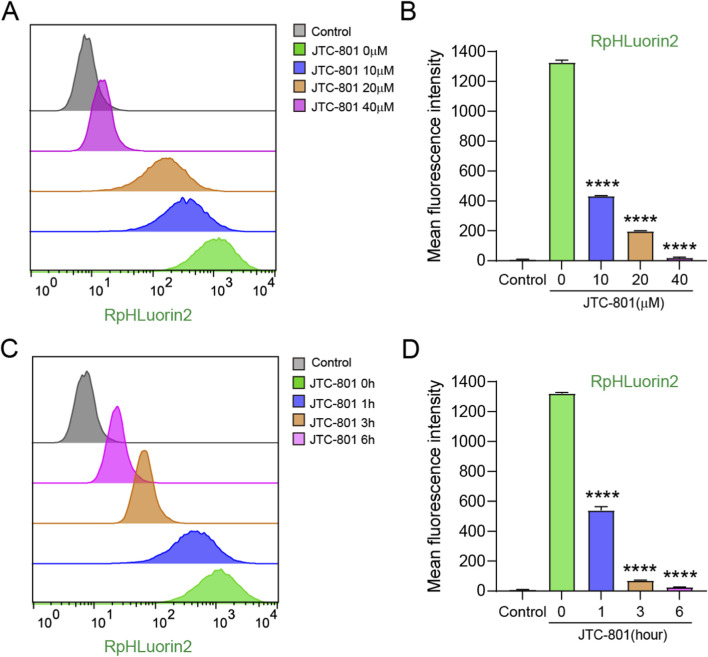
Flow cytometry analysis substantiated that JTC-801 induces dose-and time-dependent reduced RpHluorin2 fluorescence within alkaliptotic cells. **(A,B)** RpHluorin2-expressing cells were treated as Figure 2B. Cells were digested into single-cell suspensions prior to flow cytometry analysis. Flow cytometry histograms depict the distribution of RpHluorin2 fluorescence in cells under different concentrations of JTC-801 treatment. MFI value of RpHluorin2 were quantified by flowjo software. ****p < 0.0001. The MFI values obtained from other experimental groups were statistically compared with those of the 0 μM group. **(C,D)** RpHluorin2-expressing cells were treated as Figure 2C. Cells were digested into single-cell suspensions prior to flow cytometry analysis. Flow cytometry histograms showing the time-dependent changes in RpHluorin2 fluorescence distribution. MFI value of RpHluorin2 were quantified by FlowJo software. The MFI values obtained from other experimental groups were statistically compared with those of the 0 h group. ****p < 0.0001. The gray histogram represents the control group (non-expressing RpHluorin2 cells).

To further precisely characterize the dynamic changes in RpHluorin2 fluorescence during JTC-801-induced alkaliptosis, flow cytometry analyses were conducted to assess the effects of JTC-801 on alkaliptotic cells. For the dose-dependent experiment, RpHluorin2-expressing cells were treated with JTC-801 at concentrations of 10, 20, and 40 μM for 6 h, and then digested into single-cell suspensions for flow cytometry analysis. As shown in Figures 3A,B, the flow cytometry histograms revealed the distribution of RpHluorin2 fluorescence in cells under different JTC-801 concentrations. Quantification of the mean fluorescence intensity demonstrated that JTC-801 treatment dose-dependently decreased RpHluorin2 fluorescence levels compared with the untreated control group (Figure 3B). For the time-dependent experiment, cells were treated with 40 μM JTC-801 for 1, 3, and 6 h, followed by single-cell suspension preparation and flow cytometry analysis. The flow cytometry histograms (Figure 3C) showed time-dependent changes in RpHluorin2 fluorescence distribution. MFI values demonstrated that JTC-801 treatment time-dependently reduced RpHluorin2 fluorescence levels relative to the 0 h JTC-801 control group (Figure 3D). Collectively, these flow cytometry data further confirmed that JTC-801 induces a dose-and time-dependent reduction in RpHluorin2 fluorescence in alkaliptotic cells, which is consistent with the findings obtained from microplate reader and fluorescence microscopy analyses.

### 3.4 Small animal imaging system analysis reveal dose-and time-dependent reduced RpHluorin2 fluorescence in JTC-801 induced alkaliptotic cells

**FIGURE 4:**
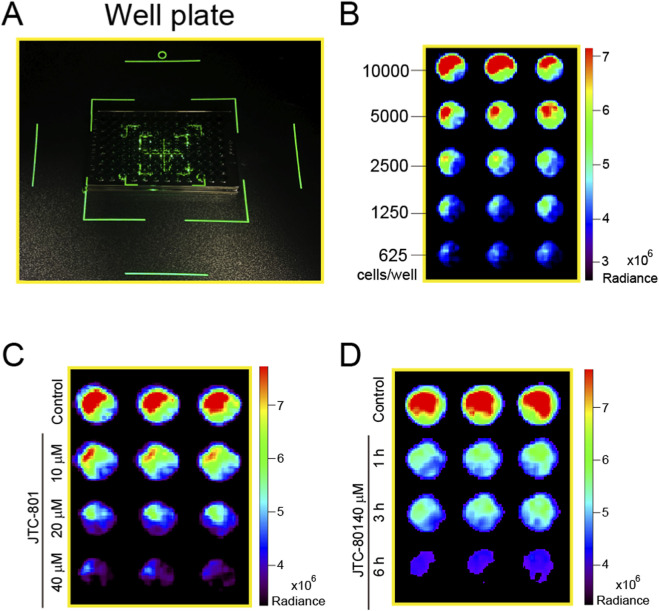
Small animal imaging system analysis confirmed that JTC-801 induces dose- and time-dependent decreases RpHluorin2 fluorescence in alkaliptotic cells. **(A)** 96-well black-walled clear-bottom microplates seeded with the cells were transferred to a small animal imaging system for fluorescence detection and image acquisition. **(B)** RpHluorin2-expressing cells were seeded at varying densities into 96-well black-walled clear-bottom microplates and incubated overnight. The fluorescence intensity was measured and recorded using small animal imaging system (IVIS Spectrum). **(C,D)** RpHluorin2-expressing cells were treated as Figures 2B,C. Fluorescence imaging was performed using an IVIS Spectrum *in vivo* imaging system equipped with a high-sensitivity cooled CCD camera, which enabled quantitative detection of RpHluorin2 fluorescence signals.

Small animal imaging systems have emerged as indispensable tools for cellular fluorescence detection research. Therefore, we further employed a small animal imaging system to detect the changes in fluorescence within RpHluorin2-expressing cells induced by JCT-801. As shown in Figure 4A 96-well black-walled clear-bottom microplates seeded with cells were transferred to a small animal imaging system for fluorescence detection and image acquisition. To examine the linear relationship between fluorescence intensity and cell number, RpHluorin2-expressing cells were seeded at varying densities into microplates and incubated overnight. Subsequently, the fluorescence intensity measured and recorded by the small animal imaging system (IVIS Spectrum) exhibited a density-related pattern (Figure 4B). For the dose-dependent analysis (Figure 4C), RpHluorin2-expressing cells treated with JTC-801 at concentrations of 10, 20, and 40 μM for 6 h showed a clear trend of decreasing RpHluorin2 fluorescence with increasing JTC-801 concentration. In the time-dependent analysis (Figure 4D), cells exposed to 40 μM JTC-801 for 1, 3, and 6 h presented a progressive reduction in RpHluorin2 fluorescence as the treatment time extended. The IVIS Spectrum *in vivo* imaging system, equipped with a high-sensitivity cooled CCD camera, enabled quantitative detection of RpHluorin2 fluorescence signals, thus providing direct evidence for the dose-and time-dependent effects of JTC-801 on RpHluorin2 fluorescence in alkaliptotic cells.

### 3.5 Protein dot blot assay is utilized to detect the fluorescence changes of RpHluorin2 in alkaliptotic cells

**FIGURE 5:**
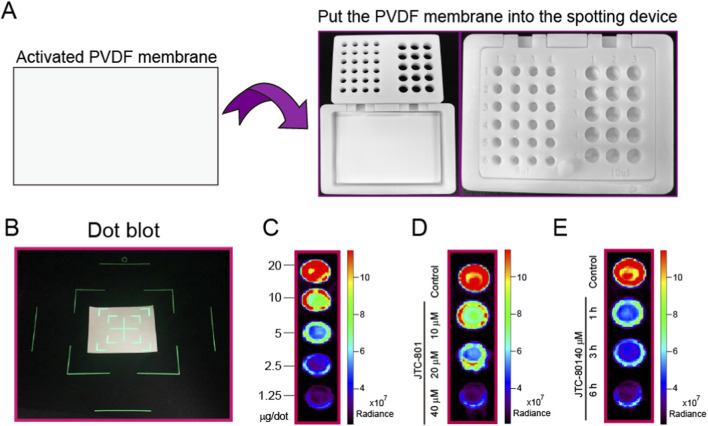
Protein dot blot assay is utilized to detect the fluorescence changes of RpHluorin2 in alkaliptotic cells. **(A)** A schematic diagram illustrating the custom-developed spotting apparatus for protein dot blot. The spotting apparatus independently developed by our laboratory. **(B)** The PVDF membranes containing the samples were transferred to a small animal imaging system for fluorescence detection and image acquisition. **(C)** Cells expressing RpHluorin2 were lysed, and varying amounts of protein were loaded according to BCA quantification. **(D)** RpHluorin2-expressing cells were treated as Figure 2B. Cells were lysed and determined by protein dot blot assay. **(E)** RpHluorin2-expressing cells were treated as Figure 2C. Cells were lysed and determined by protein dot blot assay. Fluorescence imaging was performed using an IVIS Spectrum *in vivo* imaging system equipped with a high-sensitivity cooled CCD camera, which enabled quantitative detection of RpHluorin2 fluorescence signals.

In addition to conventional intracellular fluorescence detection approaches, we innovatively developed a combined strategy that integrates a protein dot blot assay with a small animal imaging system to characterize the fluorescence dynamics of RpHluorin2 in alkaliptotic cell lysates. A custom-developed spotting apparatus (Figure 5A) was utilized to facilitate standardized sample application, thereby ensuring methodological consistency. After activating polyvinylidene difluoride (PVDF) membranes and loading samples into the proprietary device, membranes were transferred to an IVIS Spectrum imaging system for fluorescence readout (Figure 5B). Initially, RpHluorin2-expressing cells were lysed, followed by protein quantification. Subsequently, proteins at varying concentrations were spotted onto PVDF membranes to determine the sensitivity of the small animal imaging system. The results showed that a protein loading amount of 20 μg was determined to be optimal for detection (Figure 5C). For dose-response analysis, RpHluorin2-expressing cells were lysed following BCA quantification, and protein lysates were spotted onto membranes. Treatment with JTC-801 at 10, 20, and 40 μM for 6 h induced a concentration-dependent reduction in RpHluorin2 fluorescence radiance, as visualized by the graded signal attenuation across increasing JTC-801 concentrations (Figure 5D). Time-course experiments revealed progressive fluorescence loss in cells treated with 40 μM JTC-801 for 1, 3, and 6 h, with signal intensity decreasing over extended exposure (Figure 5E). The high-sensitivity cooled CCD camera integrated in the IVIS system enabled quantitative radiance measurements, confirming JTC-801 elicits dose-and time-dependent reduction of RpHluorin2 fluorescence in alkaliptotic cells. These results also indicate that RpHluorin2-expressing cells allow for the detection of fluorescence changes not only directly in intact cells but also in cell lysates via the protein dot blot assay following cell lysis providing a multi-dimensional, multi-methodological approach to investigating JTC-801-induced alkaliptosis.

## 4 Discussion

Alkaliptosis, a regulated cell death modality characterized by pH dependence and divergence from apoptosis and necroptosis, has become a compelling therapeutic target for malignant tumors, especially those that exhibit resistance to conventional therapies This is primarily attributed to the inherent pH imbalance of cancer cells and their critical reliance on maintaining pH homeostasis to sustain proliferation and facilitate metastasis (Liu et al., 2020; Chen et al., 2023a). A critical hallmark of alkaliptosis is the elevation of intracellular pH (pHᵢ), yet the lack of robust, multi-dimensional tools to monitor dynamic pHᵢ changes has hindered mechanistic investigations and the development of alkaliptosis-targeted agents. In this study, we established a stable RpHluorin2-expressing cell model and integrated five complementary fluorescence detection approaches to systematically characterize JTC-801-induced alkaliptosis, thereby filling key technical gaps in the field.

RpHluorin2, an optimized ratiometric pH-sensitive GFP variant, offers distinct advantages over traditional pH sensors: enhanced fluorescence intensity, improved pH stability, and superior folding efficiency enable non-invasive, real-time monitoring of pHᵢ dynamics in live cells (Miesenbock et al., 1998; Bizzarri et al., 2006; Mahon, 2011). Our first critical finding was the successful construction of a stable RpHluorin2-expressing 5637 cell line via lentiviral transduction. Multiple lines of evidence confirmed the reliability of this model: (1) Fluorescence imaging via a small animal imaging system (IVIS Spectrum) demonstrated robust green fluorescence emission, validating functional RpHluorin2 expression; (2) A high correlation coefficient (*R* ^2^ = 0.9998) between fluorescence intensity and cell density (Figure 1C) confirmed that fluorescence signals accurately reflect cell number, eliminating confounding variables in subsequent assays; (3) Flow cytometry clearly distinguished RpHluorin2-expressing cells from non-expressing controls (Figure 1E), confirming that RpHLuorin2 is expressed in essentially all cells. These results collectively establish RpHluorin2-expressing cells as a robust platform for pHᵢ-dependent studies, overcoming limitations of less optimized sensors (e.g., native pHluorin) that suffer from weak signal or poor pH responsiveness.

A central aim of this study was to validate whether RpHluorin2 fluorescence could report JTC-801-induced alkaliptosis, a process driven by NF-κB-mediated suppression of CA9 (a key pH regulator), leading to pHᵢ elevation. We employed four orthogonal *in vitro* detection methods, including microplate reader, fluorescence microscopy, flow cytometry, and protein dot blot, and consistently revealed dose- and time-dependent decreases in RpHluorin2 fluorescence upon JTC-801 treatment. The microplate reader assay (Figure 2B) provided high-throughput quantitative data, showing that 40 μM JTC-801 for 6 h induced the most significant fluorescence attenuation. Fluorescence microscopy corroborated these findings (Figures 2C–F) by directly visualizing reduced green fluorescence in a dose- and time-dependent manner, while nuclear staining excluded non-specific cell loss. Additionally, compared with bulk assays (e.g., microplate reader), flow cytometry captured uniformly reduction of the MFI at single-cell resolution across the cell population under JTC-801 treatment (Figures 3A–D). This consistency across methods strongly supports that RpHluorin2 fluorescence directly reports alkaliptosis progression, as the observed signal reduction aligns with the expected pHᵢ elevation (a known trigger for conformational changes in RpHluorin2 that diminish its fluorescence).

Notably, we extended our detection pipeline by incorporating two innovative approaches, which serve to enhance the application potential of this work. First, we adapted a small animal imaging system (IVIS Spectrum) for *in vitro* cell-based assays, which represents a departure from its traditional application in in vivo imaging. This system, equipped with a high-sensitivity cooled CCD camera, detected dose- and time-dependent fluorescence reductions (Figures 4C,D) with comparable sensitivity to microplate readers but offered the unique advantage of spatial resolution (e.g., well-plate mapping of fluorescence signals). This capability is critical for high-throughput screening of alkaliptosis modulators, as it enables simultaneous analysis of multiple samples while maintaining quantitative accuracy. Second, we developed a protein dot blot assay coupled with IVIS detection (Figures 5A–E), a novel method to measure RpHluorin2 in cell lysates. This approach confirmed that the JTC-801-induced reduction in fluorescence is not attributable to cell detachment or loss, but rather to cytosolic pH-dependent conformational changes in RpHluorin2, even in lysed cells. The fundamental assumption underlying this phenomenon is that the pH - responsive conformational state of RpHluorin2 is stably retained during the low-temperature lysis and membrane fixation process. During cell lysis, we used ice-cold RIPA lysis buffer supplemented with PMSF, which not only inhibits protein degradation but also minimizes temperature-induced unfolding of RpHluorin2. When spotted onto PVDF membranes and air-dried, the protein structure is fixed without altering the pH-dependent conformational state formed in intact cells. Thus, the fluorescence intensity of RpHluorin2 on the dot blot directly reflects the cytosolic pH of intact cells at the time of harvest. This mechanism is consistent with the intrinsic property of RpHluorin2, wherein intracellular alkalization (a hallmark of alkaliptosis) induces conformational rearrangements that quench its fluorescence (Mahon, 2011). Future studies will further verify this mechanism by using lysis buffers with gradient pH values, which will help to clarify the specific pH threshold for RpHluorin2 fluorescence quenching and further strengthen the reliability of this detection method. Additionally, our laboratory independently engineered a custom-developed spotting apparatus for protein dot blot assays. This apparatus ensures the standardization of spotting positions, enabling each sample to form a discrete diffusion zone. This not only effectively prevents physical mixing and cross-contamination between samples but also guarantees the accuracy of experimental results. Furthermore, the sample loading wells arranged in an array facilitate consistent spotting distances, which supports the accurate identification and localization of each sample spot by image analysis software. Consequently, this design enhances the accuracy and efficiency of data analysis while reducing the need for manual correction.

It is of paramount importance to highlight that the elevation of intracellular pH above 8.0 in RpHluorin2-expressing 5637 cells following JTC-801 treatment under 5% CO_2_ culture conditions is orchestrated by the synergistic interplay of metabolic regulation, ion transport, and bicarbonate homeostasis. From the perspective of metabolic reprogramming, cancer cells exhibit a hallmark feature of aerobic glycolysis, even under oxygen-replete microenvironments (Webb et al., 2011). Upon JTC-801 stimulation, this distinctive metabolic pattern is profoundly disrupted through the activation of NF-κB signaling, which in turn suppresses the expression of carbonic anhydrase 9 (CA9). This downregulation of CA9 interferes with the conversion of intracellular bicarbonate to carbon dioxide, concomitantly reducing intracellular acid production and thereby fostering intracellular alkalization (Tostain et al., 2010; Chen et al., 2023b; Fu et al., 2023; Chen et al., 2024). Notably, our experimental evidence demonstrates a substantial elevation in intracellular pH, an effect that is undoubtedly attributed to the combined contributions of ion transport and bicarbonate homeostasis, while the precise underlying regulatory mechanisms remain yet to be fully delineated. Collectively, metabolic regulation, ion transport, and bicarbonate homeostasis constitute an intricate regulatory network that synergistically orchestrates the aberrant elevation of intracellular pH during the process of alkaliptosis.

**FIGURE 6:**
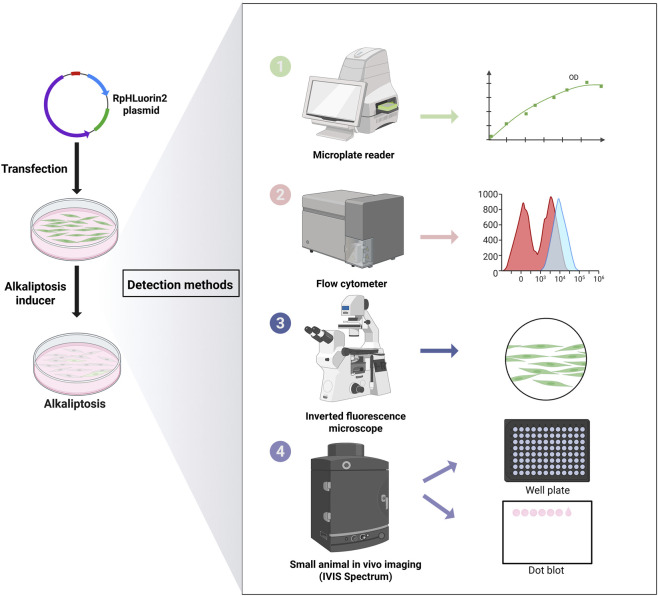
Schematic workflow for detecting alkaliptosis in RpHluorin2-expressing cells. This schematic outlines the experimantal workflow for investigating alkaliptosis in cells transfected with the RpHluorin2 plasmid. Initially, cells are transfected with the RpHluorin2 plasmid to enable fluorescent reporting. Subsequently, JTC-801, a specific agonist of alkaliptosis, is administered to induce this form of regulated cell death. Fluorescence changes associated with alkaliptosis were detected using a multi-methodological approach is employed. A microplate reader is utilized to quantify temporal changes in fluorescence intensity, as depicted in the associated kinetic plot. Flow cytometry is performed to analyze cellular fluorescence intensity distributions; the resulting histograms likely reflect differential fluorescence patterns (and thus pH-associated alterations) among alkaliptotic cell. An inverted fluorescence microscope allows for direct visual assessment, providing qualitative insights into fluorescence characteristics, as illustrated by the representative cellular image. *In vivo* imaging in small animals (IVIS Spectrum) is conducted, with readouts including well-plate-based analyses (facilitating high-throughput or spatial evaluation) and dot blots (enabling semi-quantitative assessment of fluorescence associated with cell lysis). Collectively, these approaches enable comprehensive investigation of alkaliptosis-related phenomena across multiple biological levels.

Integration of these five methods into a cohesive workflow (Figure 6) addresses a major challenge in alkaliptosis research: the lack of standardized, multi-dimensional detection assays. Previous studies have relied on single-method detection (e.g., flow cytometry alone), which may miss context-dependent nuances (e.g., spatial heterogeneity in pHᵢ). Our workflow, by contrast, enables: (1) High-throughput screening (microplate reader/IVIS); (2) Single-cell resolution (flow cytometry); (3) Visual validation (fluorescence microscopy); and (4) Biochemical confirmation (protein dot blot). This versatility makes it applicable to diverse research goals, from mechanistic studies to preclinical drug discovery (e.g., identifying novel alkaliptosis inducers). These five distinct fluorescence detection methods allow researchers to consistently select one or more approaches for their investigations, regardless of variations in laboratory settings and experimental instrumentation. This not only broadens the researchers' methodological choices but also enhances the overall applicability of the proposed methods.

While this study advances the field, several limitations should be noted. First, our model uses a single cell line (human bladder cancer 5637 cells); future work should validate RpHluorin2 expression in other cancer types (e.g., pancreatic cancer, which is particularly sensitive to alkaliptosis) to confirm generalizability. Second, we focused on JTC-801 as a prototype inducer; expanding to other alkaliptosis modulators (e.g., NF-κB agonists or CA9 inhibitors) will further validate the utility of this platform. Third, current research on alkaliptosis remains exclusively confined to the cellular level. Moving forward, concerted efforts should be directed toward developing robust and specific approaches for detecting the induction of alkaliptosis *in vivo*.

## 5 Conclusion

In summary, our studies established a stable RpHluorin2-expressing cell model and validated a multi-dimensional fluorescence detection workflow to monitor JTC-801-induced alkaliptosis. The consistency of dose- and time-dependent fluorescence reductions across microplate reader, microscopy, flow cytometry, IVIS imaging, and protein dot blot assays confirms the reliability of this detection platform. By providing a standardized and versatile toolset, this work not only facilitates mechanistic investigations into alkaliptosis and accelerates the development of novel pH-targeted cancer therapies, but also opens new avenues for exploring fundamental biological mechanisms and dissecting disease-associated pH dysregulation.

## Data Availability

The original contributions presented in the study are included in the article/Supplementary Material, further inquiries can be directed to the corresponding authors.
