# Supplementary figures and images for: Establishment of RpHluorin2-expressing cell and its application in monitoring JTC-801-induced alkaliptosis via multi-dimensional fluorescence detection approaches
Source: Front Cell Dev Biol. 2025 Dec 12;13:1727740. doi: 10.3389/fcell.2025.1727740 (PMC12741124; doi:10.3389/fcell.2025.1727740)

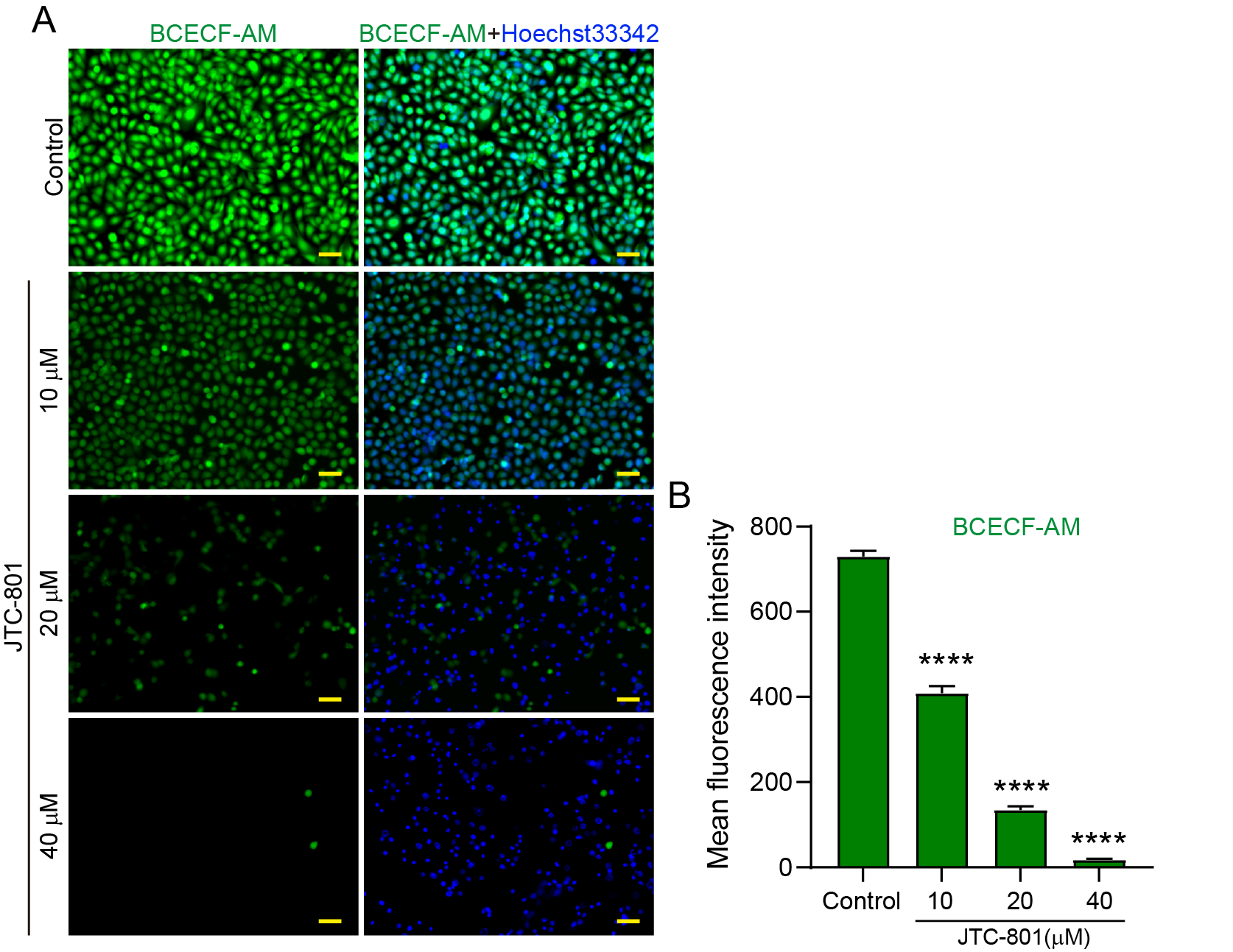

Supplement: Supplementary file 1 [file Image2.tif]

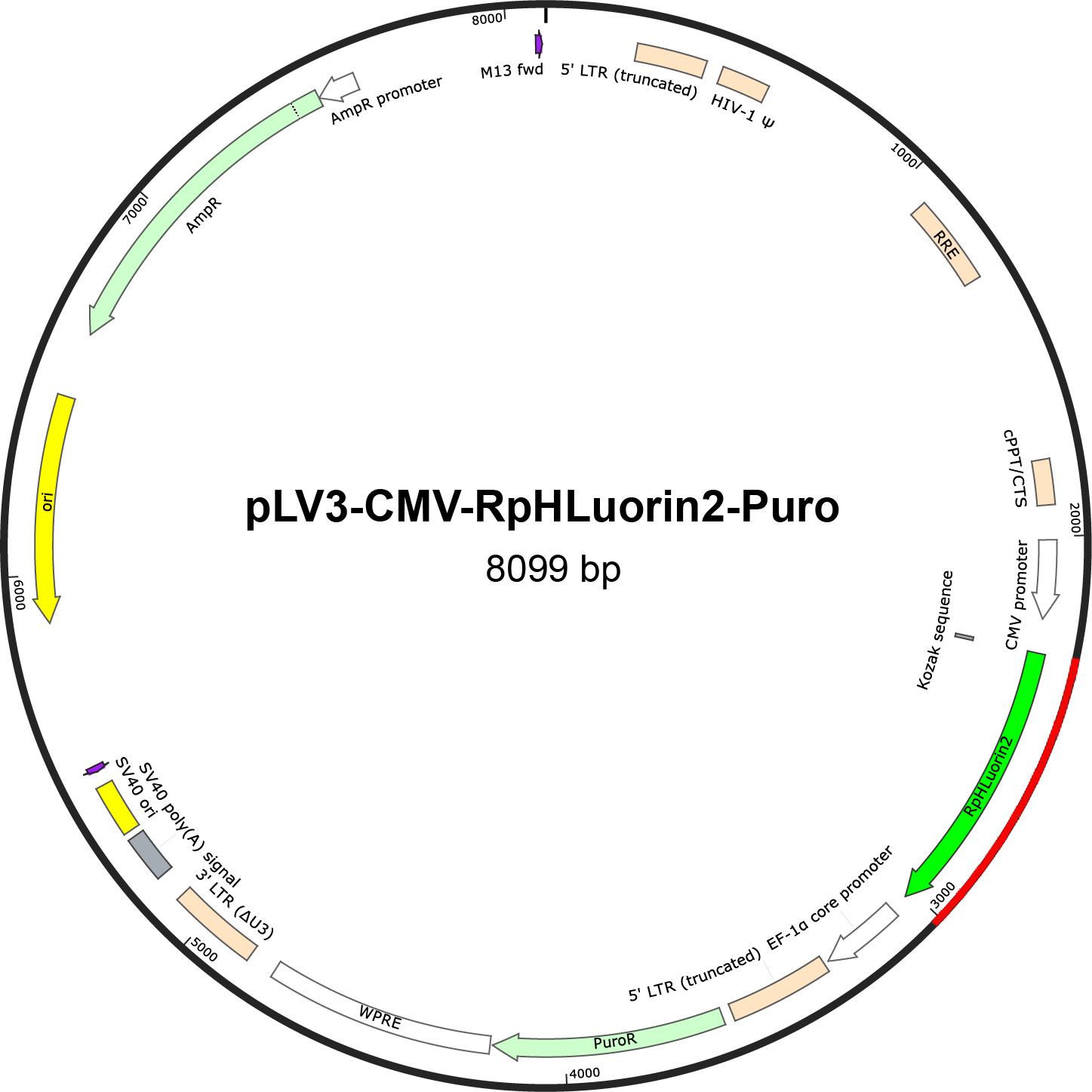

Supplement: Supplementary file 2 [file Image1.tif]
